# The Influence of Natural Head Position on the Cervical Sagittal Alignment

**DOI:** 10.1155/2017/2941048

**Published:** 2017-08-13

**Authors:** Kuan Wang, Zhen Deng, Zhengyan Li, Huihao Wang, Hongsheng Zhan

**Affiliations:** ^1^Shi's Center of Orthopedics and Traumatology, Shuguang Hospital Affiliated to Shanghai University of TCM, Shanghai 201203, China; ^2^Tongji Hospital, Tongji University School of Medicine, Shanghai 200092, China; ^3^Baoshan Branch, Shuguang Hospital Affiliated to Shanghai University of TCM, Shanghai 201900, China; ^4^Institute of Traumatology & Orthopedics, Shanghai Academy of TCM, Shanghai 201203, China

## Abstract

**Introduction:**

This study investigated the relationship between the parameters related to the natural head position and cervical segmental angles and alignment of patients with neck pain.

**Material and Methods:**

The lateral radiographs of the cervical spine were collected from 103 patients and were used to retrospectively analyze the correlation between the natural head position, cervical local sagittal angles, and alignment. Sagittal measurements were as follows: cervical curvature classification, slope of McGregor's line (McGS), local sagittal angles (C0–C2 angle, C2–C5 angle, C5–C7 angle, and C2–C7 angle), T1 slope, center of gravity of the head to sagittal vertical axis (CG–C7 SVA), and local sagittal alignment (C0–C2 SVA and C2–C7 SVA).

**Results:**

McGS was significantly correlated to C0–C2 angle (*r* = 0.57), C0–C2 SVA (*r* = −0.53), C2–C7 SVA (*r* = −0.28), and CG–C7 SVA (*r* = −0.47). CG–C7 SVA was also significantly correlated to curvature type (*r* = 0.27), C5–C7 angle (*r* = −0.37), and C2–C7 angle (*r* = −0.39).

**Conclusions:**

A backward shift with an extended head position may accompany a relatively normal curvature of the cervical spine. The effect of posture control in relieving abnormal mechanical state of the cervical spine needs to be further confirmed by biomechanical analysis.

## 1. Introduction

Cervical curvature is one of the clinical assessments which could reflect the mechanical state of the cervical spine. With a normal lordotic curvature, the least amount of energy is spent to maintain the horizontal gaze in the upright position [1]. Under this condition, tensile and compression loads on spinal structures are smallest compared to other cervical alignments [2, 3].

The cervical spine connects the skull and thoracic vertebrae and supports the mass of the head. It is a multijoint structure that allows complex movement of the neck. In recent years, many studies have used X-ray imaging to explore the relationship between parameters related to cervical sagittal alignment, involving the mass point of the head, C2 vertebral center, T1 slope, and other measurements [4]. Lee et al. found that T1 slope and C2–C7 Cobb angle were strongly correlated [5], whereas the cranial offset and C2–C7 Cobb angle were in moderate correlation. Nunez-Pereira et al. found that the occiput–C2 angle and C2–C7 Cobb angle also showed a moderate correlation [6]. Tang et al. found a close relationship between the C2–C7 sagittal vertical axis (C2–C7 SVA) and health-related quality of life in patients who underwent surgery of the cervical spine [7].

The above studies have shown that the segmental angle, alignment of the cervical spine, and the position of the first thoracic spine were interdependent and may contribute to the progression of cervical degeneration and quality of life. These studies focused a lot on the effect of cervicothoracic junction towards sagittal alignment of the cervical spine, but less attention was paid to the head position. As already known, the head position could be controlled freely by our mind in a certain range of motion, and it needs a little moment to move the head in a neutral zone [8]. For a proper transmission of the head weight, there should be some relationship between the position of the head in the natural standing position, the local sagittal angles, and the alignment of the cervical spine. The natural head position in the sagittal plane may include the rotation and translation degrees of freedom, which could be represented by a head tilt and forward/backward head shift [9] or by McGregor slope (McGS) and C0–C7 sagittal vertical axis (CG–C7 SVA) [10, 11]. We hypothesized that these parameters of the head position have some impacts on the cervical segmental angles and alignment.

Therefore, the objective of this study was to investigate the relationship between the parameters related to the natural head position and the cervical alignment, through retrospectively analyzing the cervical lateral radiographs of 103 patients with neck pain.

## 2. Materials and Methods

### 2.1. Study Design

This study was a retrospective data analysis of consecutive patients who underwent sagittal plane cervical spine X-ray from January 2015 to January 2016. Inclusion criteria were adult neck pain patients (>18 years of age), with symptoms like neck stiffness, pain, and local tenderness, with or without limited cervical activity. Their X-ray examination can be normal or with mild degeneration. Exclusion criteria included patients with ankylosing spondylitis, tumor, cervical fracture or dislocation, diffuse idiopathic bone hypertrophy, and other specific diseases or mutations.

### 2.2. Radiographic Analysis

**Figure 1:**
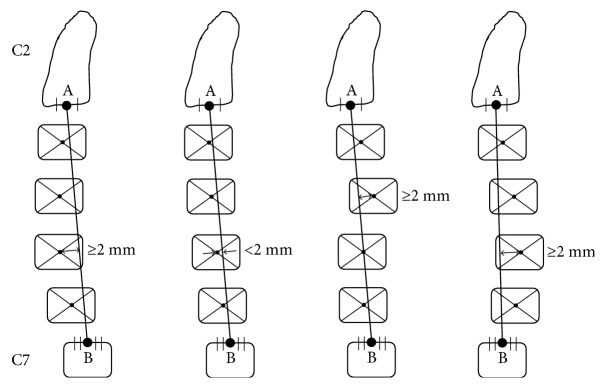
The cervical curvature types (from left to right: lordosis, straight, sigmoid, and kyphosis).

**Figure 2:**
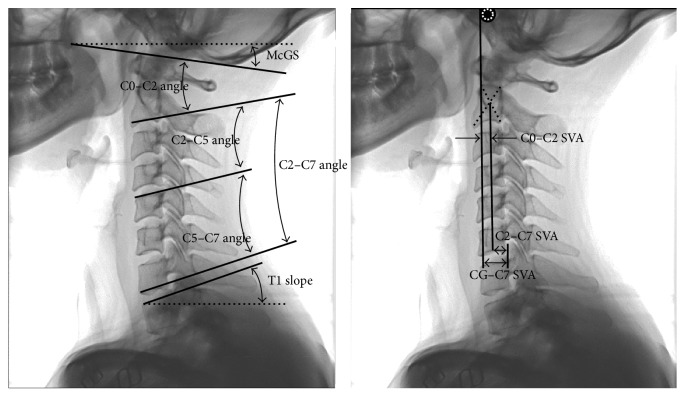
The measurement of parameters related to natural head position and cervical alignment (McGS, slope of McGregor's line; SVA, sagittal vertical axis).

The lateral radiographs of the cervical spine were taken when patients were in the standing position. The measurements included cervical curvature classification (Figure 1) and parameters related to the head position and cervical alignment (Figure 2). The entire measurements included the following parameters:

1. Cervical curvature classification [12]: the cervical curvature was divided into three types including lordosis, straight/sigmoid, and kyphosis as types 1–3. To classify the curvature, a line was drawn to connect the midpoint of the C2 inferior end plate and C7 superior end plate. Then, the center of each vertebral body from C3 to C6 was located by connecting the diagonals of each vertebral body in the sagittal plane, and the distance from each center to line AB (see Figure 1) was measured. If all centers of vertebral bodies were in front of the line and the maximum distance was greater than 2 mm, the curvature was classified into the lordosis group; if the centers were in front of or behind the line but the maximum value of distance was no more than 2 mm, the curvature was classified into the straight group. For the sigmoid group, the centers were in front of or behind the line, but the maximum value of distance was greater than 2 mm. For the kyphosis group, the centers were all behind the line connecting the C2–C7 endplates, and the maximum value of distance was greater than 2 mm.
2. Slope of McGregor's line (McGS) [13]: McGregor slope was defined as the slope of the line that connected the posterior margin of the hard palate and foramen magnum against the horizontal plane. This value was reported to be strongly correlated with the chin-brow vertical angle (CBVA) (*r* = 0.862) which was one measurement of horizontal gaze. So this value was used in the current study as an angular assessment of the natural head position. A positive value means that the head was in the extension position with ascending gaze, and a negative value means that the head was in the flexion position with descending gaze.
3. Local sagittal angles: local sagittal angles included C0–C2 angle, C2–C5 angle, C5–C7 angle, and C2–C7 Cobb angle. C0–C2 angle was defined as the angle between the inferior endplates of C2 and McGregor's line [6]. C5–C7 angle denoted the angle between the C5 superior endplate and C7 inferior endplate [14]. C2–C7 Cobb angle denoted the angle between the C2 inferior endplate and C7 inferior endplate [5]. In the current study, to see the effect of the head position on the middle cervical spine, C2–C5 angle was defined as the value of C2–C7 Cobb angle subtracted by C5–C7 angle.
4. T1 slope [10]: T1 slope was defined as the angle between the line that was parallel to the superior end plate of T1 and the horizontal plane.
5. The center of gravity of the head to sagittal vertical axis (CG–C7 SVA): CG–C7 SVA was defined as the distance between a plumb line dropped from the anterior margin of the external auditory canal and the posterior superior corner of C7. This value reflected relative translation of the head against the C7 vertebra [10].
6. Local sagittal alignment: C2–C7 sagittal vertical axis (C2–C7 SVA) was defined as the distance between a plumb line dropped from the centroid of C2 and the posterior superior corner of C7 [7]. This value reflected the relative translation of the C2 vertebra against the C7 vertebra. To see the effect of the head position on the sagittal alignment of the upper cervical spine, we defined the C0–C2 sagittal vertical axis (C0–C2 SVA) as the value of CG–C7 SVA subtracted by C2–C7 SVA.

The above parameters concerning the head position and cervical alignment have been widely used in previous studies with good reliability [10]. These measurements were processed independently by two experienced radiologists, and the measured values were averaged to produce the results. The study protocol was approved by the medical ethics committee of our hospital.

To investigate the effect of the natural head position on the cervical alignment, the subjects were divided into four groups according to their CG–C7 SVA and McGS values. The median CG–C7 SVA was used for the division of the forward shift head position or backward shift head position. Lafage el al. reported the mean value of McGS and equations generated with linear regression between CBVA and McGS [13]. According to their results, 0.87° was the mean value of McGS, and this value was used for the division of the extended or flexed cranial position. Therefore, in terms of translation and rotation of the head position in the sagittal plane, four groups included forward shift with the extended head position (FE), backward shift with the extended head position (BE), forward shift with the flexed head position (FF), and backward shift with the flexed head position (BF).

### 2.3. Statistical Analysis

Data were analyzed by SPSS 20.0 software. Values were presented as mean ± SD. Spearman's correlation analyses were performed to examine associations among the selected variables. The curvature type numbers 1–3 were treated as ordinal categorical variables in the current study. If the curvature type number is negatively correlated with a local segmental angle, an increase in the curvature type number is correlated with a decrease in the angle. This results should be interpreted as that the curvature type number would be more close to 1 (lordosis type) rather than 3 (kyphosis type), with a decrease in the local segmental angle. The comparison of each radiographic parameter among four head positions was determined by using one-way ANOVA with Bonferroni's post hoc test. A *P* value equal to or less than 0.05 was set up as threshold for statistical significance.

## 3. Results

### 3.1. Demographic and Cervical Curvature Classification

There were 103 subjects included in this study. Among these subjects, 33 subjects were male and 70 were female. The mean age was 37.4 ± 12.3 years. There were 35 subjects allocated to the lordosis group, 56 subjects to the straight or sigmoid group (only one subject was classified as sigmoid curvature; thus, the two groups were combined), and 12 subjects to the kyphosis group. The mean age of each group was 39.4 ± 13.2, 37.3 ± 11.4, and 32.7 ± 13.6 years, respectively. There was no significant difference in age and gender between each group (*P* > 0.05).

### 3.2. Measurement of Parameters and Correlation Analysis

The results of radiographic measurements related to the head position and cervical alignment are shown in Table 1. The results of Spearman's correlation analyses are shown in Table 2. McGS was found to be significantly correlated to C0–C2 angle (*r* = 0.57), C0–C2 SVA (*r* = −0.53), C2–C7 SVA (*r* = −0.28), and CG–C7 SVA (*r* = −0.47) but not significantly correlated to curvature classification, C2–C5 angle, C5–C7 angle, and C2–C7 angle. CG–C7 SVA was also significantly correlated to curvature type (*r* = 0.27), C5–C7 angle (*r* = −0.37), and C2–C7 angle (*r* = −0.39).

### 3.3. The Influence of Different Head Positions

**Figure 3:**
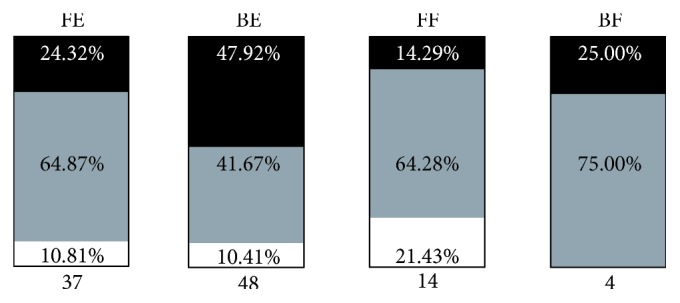
The proportion of each cervical curvature type and total number of subjects in different head position (FE, forward shift with extended head position; BE, backward shift with extended head position; FF, forward shift with flexed head position; BF, backward shift with flexed head position; black square, lordosis; gray square, straight or sigmoid; white square, kyphosis).

Figure 3 shows the distribution of cervical curvatures in different head positions. Among the four groups (FE, BE, BF, and FF groups), the proportion of subjects with lordosis curvature in the BE group was most compared to those in other groups, and the proportion of subjects with straight or sigmoid curvature was most in the FE group. In the FF group, the proportion of subjects with kyphosis curvature was most compared to those in other groups. There were only four subjects in the BF group, with three subjects classified with straight/sigmoid curvatures.  Table 3 shows the comparison of each radiographic parameter among four head positions determined by one-way ANOVA with Bonferroni's post hoc test. C0–C2 angle, C5–C7 angle, C2–C7 angle, C0–C2 SVA, and C2–C7 SVA are significantly different among the four head positions. C2–C5 angle and T1 slope are not significantly different among the four head positions.

## 4. Discussion

In this study, the lateral radiographs of the cervical spine were used to analyze the relationship between the natural head position and cervical alignment. The measured parameters related to cervical alignment (e.g., C2–C7 angle and T1 slope) were consistent with the reported data [5, 6, 12]. This study provides some insight into the effect of the head position on the cervical spine alignment.

Since T1 was the fixed end of the cervical spine and the cervical column is influenced by the weight of the head, it is expected that the T1 slope might play a determining role in the curvature of the cervical spine. In the current study, T1 slope was in positive correlation with C2–C7 angle. The results agreed with the data reported by Lee et al. [5].

The translation or rotation of the head is controlled by the nervous system to keep an economic posture [14]. Due to the large vision field, people could maintain a horizontal gaze with varying head positions within a certain range. In the current study, the CG–C7 SVA was found to be in negative correlation with curvature type. This means that it is more likely to be kyphotic of the cervical spine with the anterior translation of the head. Furthermore, the CG–C7 SVA was found to be negatively correlated with C5–C7 angle and C2–C7 angle but not significantly correlated with C2–C5 angle. This result suggested that a forward shift of the head in the natural head position might indicate a loss of lordosis in the lower cervical spine.

This study also found that the extended or flexed position of the head represented by McGS was not correlated with local sagittal angle except C0–C2 angle. This might be because most flexion and extension range of motion is found at C0–C2 compared to mid and lower cervical regions [8]. Thus, it has a great compensatory space for the flexion/extension of the head and minimizes the impact on the local segmental angle below C2. Additionally, the McGS was found to be in negative correlation with C0–C2 SVA, C2–C7 SVA, and CG–C7 SVA, which means that the mass point of the head moves posteriorly with the extension of the head. The results suggested that the rotation in the natural head position might be indirectly involved in the balance control of the cervical spine by adjusting the translation of the head. A study by Tang et al. found that patients who underwent posterior cervical fusion surgery reported a decrease in the quality of life with an increased value of C2–C7 SVA [7]. According to our results, the anterior translation of the head might accompany cranial flexion as well as a flattening of the cervical lordosis. To balance the extensor moment produced by the flexed head, the extensor muscles need to produce additional extension torque and the fatigue of these muscles might lead to neck or upper back symptoms. Therefore, it might be more appropriate to keep the head in a neutral position rather than a flexed positon to prevent neck and back pain.

With the development of modern electronic devices, a forward head posture is common among people [15] and the forward head posture has been associated with musculoskeletal pain in previous studies [16, 17]. Although the total number of subjects with kyphosis curvature was small in the current study, the proportion of subjects with the kyphosis type curvature was most in the FF group. This result suggested that the forward shift with the flexed head position may be associated with cervical kyphosis thus leading to the unbalance of a mechanical state. In contrast, the proportion of the lordosis type was most in the BE group, meaning that a backward shift with an extended head position was more likely to be accompanied with a normal cervical curvature. The cervical spine could be divided into three columns (one vertebral body and two facet joints on the same level) [10]. With the backward shift and the extended head position, the weight of the head would be more loaded on the posterior column of the cervical spine. Since we found that C2–C5 angle was not significantly different among the four head positions, this position (BE) might relieve the load on the intervertebral disc of C5–C7 segments and be helpful in compensating the additional load caused by nonphysiological cervical curvature like kyphosis and straight/sigmoid type. In the current study, some subjects with kyphotic curvature were found to have a forward shift and flexed position of the head. Whether posture control (e.g., keeping a backward shift and extended head position) is helpful to these subjects in relieving abnormal mechanical state of the cervical spine needs to be further confirmed by biomechanical analysis.

Some limitations existed in this study. Since this study was a retrospectively radiograph analysis, it could not accurately reflect the causal relationship between each parameter and no healthy subjects as the control group. Age is also an important factor affecting the curvature. In the current study, there is a trend that the age of subjects with nonphysiological cervical curvature (straight, sigmoid, and kyphosis) become younger. Also, the cervical curvature in male and female showed a different trend with increasing age [10]. Further prospective study including measurements of the quality of life and pain in terms of age, along with the neurological deficits and MRI images, which could accurately show the degeneration of intervertebral disc, would uncover the causal relationship of them.

## 5. Conclusions

The natural head position was related to local sagittal angle and alignment of the cervical spine. The mass point of the head moved posteriorly with the extension of the head. Backward shift with an extended head position may accompany a relatively normal curvature of the cervical spine. Some subjects with nonphysiological cervical curvature were in the forward shift and flexed head position. The effect of posture control in relieving abnormal mechanical state of the cervical spine needs to be further confirmed by biomechanical analysis.

## Figures and Tables

**Table 1:** Radiographic measurements related to natural head position and cervical alignment.

Variable	*N*	Min	Max	Mean	SD
McGS (°)	103	−12.00	20.90	6.14	6.10
C0–C2 angle (°)	103	0.80	34.80	16.61	7.39
C2–C5 angle (°)	103	−19.70	21.50	2.88	7.71
C5–C7 angle (°)	103	−11.80	17.30	4.52	6.61
C2–C7 angle (°)	103	−16.80	30.30	7.40	9.55
T1 slope (°)	103	8.70	43.40	23.31	6.64
C0–C2 SVA (mm)	103	−16.50	22.30	1.36	6.77
C2–C7 SVA (mm)	103	−4.60	49.80	17.91	8.54
CG–C7 SVA (mm)	103	−12.00	72.10	19.27	13.12

McGS: slope of McGregor's line; SVA: sagittal vertical axis.

**Table 2:** The correlation analysis between parameters.

Variable		Curvature type	McGS	C0–C2 angle	C2–C5 angle	C5–C7 angle	C2–C7 angle	T1 slope	C0–C2 SVA	C2–C7 SVA	CG–C7 SVA
Curvature type	*r*	1	−0.11	**0.38**	**−0.61**	**−0.33**	**−0.73**	**−0.40**	**0.42**	0.06	**0.27**
	*P*		0.29	**<0.01**	**<0.01**	**<0.01**	**<0.01**	**<0.01**	**<0.01**	0.55	**0.01**
McGS	*r*		1	**0.57**	0.05	0.08	0.12	−0.05	**−0.53**	**−0.28**	**−0.47**
	*P*			**<0.01**	0.63	0.42	0.24	0.59	**<0.01**	**<0.01**	**<0.01**
C0–C2 angle	*r*			1	**−0.51**	−0.17	**−0.53**	−0.07	0.12	0.18	0.18
	*P*				**<0.01**	0.08	**<0.01**	0.46	0.24	0.07	0.07
C2–C5 angle	*r*				1	−0.12	**0.69**	**0.29**	**−0.46**	0.14	−0.16
	*P*					0.24	**<0.01**	**<0.01**	**<0.01**	0.15	0.11
C5–C7 angle	*r*					1	**0.58**	**0.48**	**−0.22**	**−0.38**	**−0.37**
	*P*						**<0.01**	**<0.01**	**0.03**	**<0.01**	**<0.01**
C2–C7 angle	*r*						1	**0.55**	**−0.53**	−0.15	**−0.39**
	*P*							**<0.01**	**<0.01**	0.13	**<0.01**
T1 slope	*r*							1	−0.05	**0.28**	0.13
	*P*								0.61	**<0.01**	0.21
C0–C2 SVA	*r*								1	**0.39**	**0.79**
	*P*									**<0.01**	**<0.01**
C2–C7 SVA	*r*									1	**0.85**
	*P*										**<0.01**
CG–C7 SVA	*r*										1
	*P*										

Note: bold font indicates statistical significance (*P* < 0.05). McGS: slope of McGregor's line; SVA: sagittal vertical axis.

**Table 3:** ANOVA test for comparison of each radiographic parameter among four head positions.

Variable	Head position				*P*
	FE	BE	FF	BF	
C0–C2 angle	19.38 ± 8.05	16.38 ± 6.50	12.86 ± 4.92★	6.85 ± 5.09★	0.001
C2–C5 angle	1.13 ± 7.78	4.51 ± 7.97	2.51 ± 6.56	0.88 ± 5.19	0.227
C5–C7 angle	3.23 ± 6.05	6.39 ± 6.76	0.75 ± 5.62▲	7.15 ± 6.33	0.013
C2–C7 angle	4.36 ± 8.09	10.90 ± 9.82★	3.26 ± 8.61▲	8.03 ± 9.66	0.004
T1 slope	23.67 ± 6.02	23.10 ± 7.62	23.54 ± 5.29	21.70 ± 5.06	0.940
C0–C2 SVA	4.74 ± 5.47	−3.40 ± 4.74★	8.41 ± 4.75▲	2.65 ± 3.78	<0.001
C2–C7 SVA	22.21 ± 5.32	12.71 ± 6.39★	26.93 ± 8.98▲	9.10 ± 3.12★■	<0.001

Note: ★ indicates *P* < 0.05 in comparison with the FE group; ▲ indicates *P* < 0.05 in comparison with the BE group; ■ indicates *P* < 0.05 in comparison with the FF group.
